# Late Observations and Studies on Traumatic Fevers

**Published:** 1872-12

**Authors:** 


Late Observations and Studies on Traumatic Fever.—The celebrated surgeon, Dr. Billroth, of Vienna, has lately again returned to his former researches on traumatic fever, and has executed a long series of difficult experiments published in *Langenbeck's Archiv.* xiii, No. 3, partly for the correction of errors and the completion of deficiencies in his former labors, partly for the consideration of late views and ideas which have sprung up in relation to this subject.

Persistent measurements of the rectal temperature in dogs in the normal state, were made, the influence of injuries and operations relatively on dogs and men in the first few hours afterwards, and that of muscular exertion on the same were defined by careful and tedious measurements of the bodily temperature. In relation to the origin of fever, measurements determining the influence of permanent mechanical and chemical irritation of sensitive nerves on the bodily temperature, and on the vaso-motor nerves were made, and at last experiments about the creation of fever by the Injection of good and ill-conditioned pus, distilled water, serum of blood, etc., were repeated and enlarged.

The different series of separate experiments establish principally the following facts: 1. A constant accumulation of caloric in the body takes place in consequence of muscular exertions, and of injections of unhealthy pus in certain quantities. 2. Simple and uncomplicated wounds and different irritations of the vaso-motor nerves do not occasion any fever or increase of bodily temperature in the first three hours after the injury or irritation has been sustained, while emboli in the pulmonary arteries, and the injection of water and healthy pus cause fever at once.

Billroth decides with Liebermeister that fever is not caused by diminished radiation of caloric, but by increased production of it within the body, and holds that this surplus production in traumatic fever is produced by the introduction of substances into the blood from the seat of the inflammation or gangrene, which deranges the controlling influence of the central nervous system over the regulation of the bodily temperature. He does not admit any difference between the production of inflammation or decomposition, and believes that the fever produced is the direct consequence of the introduction of those deleterious substances in the blood from the seat of the inflammation or gangrene, but not indirectly that of an inflammation previously established by them somewhere within the organism.

The views of Hueter and Klebs, that microscopic organisms cause the decomposition of the wounds, as well as the traumatic fever, he does not consider sufficiently proven to look upon them as anything more than strong possibilities.—*Berliner Klinische Wochenschrift, No.* 38, 1872.

